# The Next-Generation β-Lactamase Inhibitor Taniborbactam Restores the Morphological Effects of Cefepime in KPC-Producing Escherichia coli

**DOI:** 10.1128/Spectrum.00918-21

**Published:** 2021-09-08

**Authors:** Elyse J. Roach, Tsuyoshi Uehara, Denis M. Daigle, David A. Six, Cezar M. Khursigara

**Affiliations:** a Department of Molecular and Cellular Biology, University of Guelph, Guelph, Ontario, Canada; b Venatorx Pharmaceuticals, Inc., Malvern, Pennsylvania, USA; University of Arizona/Banner Health

**Keywords:** antibiotics, resistance, bacteria, microscopy, susceptibility

## Abstract

Gram-negative bacteria producing carbapenemases are resistant to a variety of β-lactam antibiotics and pose a significant health risk. Given the dearth of new antibiotics, combinations of new broad-spectrum β-lactamase inhibitors (BLIs) with approved β-lactams have provided treatment options for resistant bacterial infections. Taniborbactam (formerly VNRX-5133) is an investigational BLI that is effective against both serine- and metallo-β-lactamases, including the serine carbapenemase KPC. In the current study, we assessed the effectiveness of taniborbactam to restore antibacterial activity of cefepime against KPC-3-producing Escherichia coli by inhibiting the KPC-3-dependent hydrolysis of cefepime. Time-lapse microscopy revealed that cells treated with greater than 1× MIC of cefepime (128 μg/ml) and cefepime-taniborbactam (4 μg/ml cefepime and 4 μg/ml taniborbactam) exhibited significant elongation, whereas cells treated with taniborbactam alone did not owing to a lack of standalone antibacterial activity of the BLI. The elongated cells also had frequent cellular voids thought to be formed by attempted cell divisions and pinching of the cytoplasmic membrane. Additionally, the effect of taniborbactam continued even after its removal from the growth medium. Pretreatment with 4 μg/ml taniborbactam helped to restore the antibacterial action of cefepime by neutralizing the effect of the KPC-3 β-lactamase.

**IMPORTANCE** β-lactam (BL) antibiotics are the most prescribed antimicrobial class. The efficacy of β-lactams is threatened by the production of β-lactamase enzymes, the predominant resistance mechanism impacting these agents in Gram-negative bacterial pathogens. This study visualizes the effects of a combination treatment of taniborbactam, a broad spectrum β-lactamase inhibitor (BLI), and the BL antibiotic cefepime on a carbapenemase-producing E. coli strain. While this treatment has been described in the context of other cephalosporin-resistant bacteria, this is the first description of a microscopic evaluation of a KPC-3-producing strain of E. coli challenged by this BL-BLI combination. Live-cell microscopy analysis of cells treated with taniborbactam and cefepime demonstrated the antimicrobial effects on cellular morphology and highlighted the long-lasting inhibition of β-lactamases by taniborbactam even after it was removed from the medium. This research speaks to the importance of taniborbactam in fighting BL-mediated antibiotic resistance.

## INTRODUCTION

Antibiotic resistance is a growing concern when treating bacterial infections. Not only is the number of resistance mechanisms acquired by bacteria increasing each year but the prevalence of multidrug-resistant (MDR) bacteria is also increasing (1). Few treatment options remain as the rate of discovery and development of new antibiotics is outpaced by bacterial resistance development (2). Importantly, β-lactam (BL) antibiotics have long been the front-line treatment for bacterial infections, but the widespread production of β-lactamases continues to compromise this first line of defense. Thus, development of β-lactamase inhibitors (BLIs) to combine with β-lactams is an essential strategy to combat MDR bacteria (2). The amoxicillin-clavulanate combination has been used clinically for decades (3). Recently, more BL-BLI combinations, such as ceftazidime-avibactam and meropenem-vaborbactam, have been approved to treat infections by MDR Gram-negative bacteria producing a wide variety of serine-β-lactamases (2–4). However, vaborbactam lacks activity against OXA carbapenemases, and neither BLI is active against metallo-β-lactamases (5).

Cefepime-taniborbactam is a novel BL-BLI combination currently in clinical development to treat complicated urinary tract infections associated with MDR bacterial pathogens producing carbapenemases (6–8). Cefepime, a fourth-generation cephalosporin, inhibits growth of susceptible bacteria by covalently binding to penicillin-binding proteins (PBP), mainly PBP3 that is essential for septal cell wall synthesis (9). Inhibition of PBP3 effectively halts cross-linking of newly synthesized peptidoglycan polymers at the cell division site while the growing bacterial cell forms long filaments and continues to remodel septal peptidoglycan by hydrolysis for cellular division (9, 10). This remodeling, in the absence of PBP3 activity, leads to foci of cell wall weakness, cell bulging, and ultimately cell lysis (11, 12). Taniborbactam (formerly VNRX-5133) is a cyclic boronate BLI that inhibits clinically relevant serine- and metallo-β-lactamases (6–8), including extended-spectrum β-lactamases and carbapenemases, such as KPC-2, KPC-3, OXA-48, NDM-1, VIM-2, and SPM-1 (13, 14). Taniborbactam exhibits reversible covalent inhibition of serine-β-lactamases and competitive inhibition of dizinc active site subclass B1 metallo-β-lactamases, blocking cefepime hydrolysis and restoring the activity of the β-lactam against MDR Gram-negative bacteria (6, 7).

This study aimed to evaluate the impacts and consequences of the addition of taniborbactam on cefepime-elicited morphological effects using KPC-3-producing Escherichia coli. We describe the bactericidal activity of cefepime-taniborbactam and morphological effects upon treatment. We also examined the lifetime of β-lactamase inhibition originating from pretreatment with taniborbactam using time-lapse microscopy. Our results suggest that lysis caused by cefepime while protected by taniborbactam is indistinguishable from that observed with cefepime alone and that pretreatment of cells with taniborbactam results in a prolonged cefepime protective effect to maintain antibacterial activity.

## RESULTS AND DISCUSSION

### Susceptibility of KPC-3-producing E. coli to cefepime and taniborbactam.

To examine potentiation of cefepime antibacterial activity by taniborbactam, we used E. coli CDC-0001 producing the KPC-3 carbapenemase. Broth microdilution assays were performed to determine MICs of antibiotics against E. coli CDC-0001 (Table 1). The modal MIC for cefepime against CDC-0001 from 3 biological replicates was 128 μg/ml, consistent with Antibiotic Resistance (AR) Isolate Bank reporting (>32 μg/ml). Taniborbactam did not inhibit growth of CDC-0001 even at 128 μg/ml when tested alone, but it did potentiate the antibacterial activity of cefepime to an MIC of 4 μg/ml when tested at a fixed BLI concentration of 4 μg/ml. Attenuated growth of E. coli CDC-0001 was also observed at sub-MICs of taniborbactam-protected cefepime (Fig. S1 in the supplemental material).

**TABLE 1:** MICs determined for E. coli CDC-0001

Compound	MICs (μg/ml) (*N* = 3)		
	Range	Median	Mode
Cefepime	64–128	128	128
Cefepime + 4 μg/ml taniborbactam	4	4	4
Taniborbactam	>128	>128	>128

### Taniborbactam restores the bactericidal activity of cefepime.

**FIG 1:**
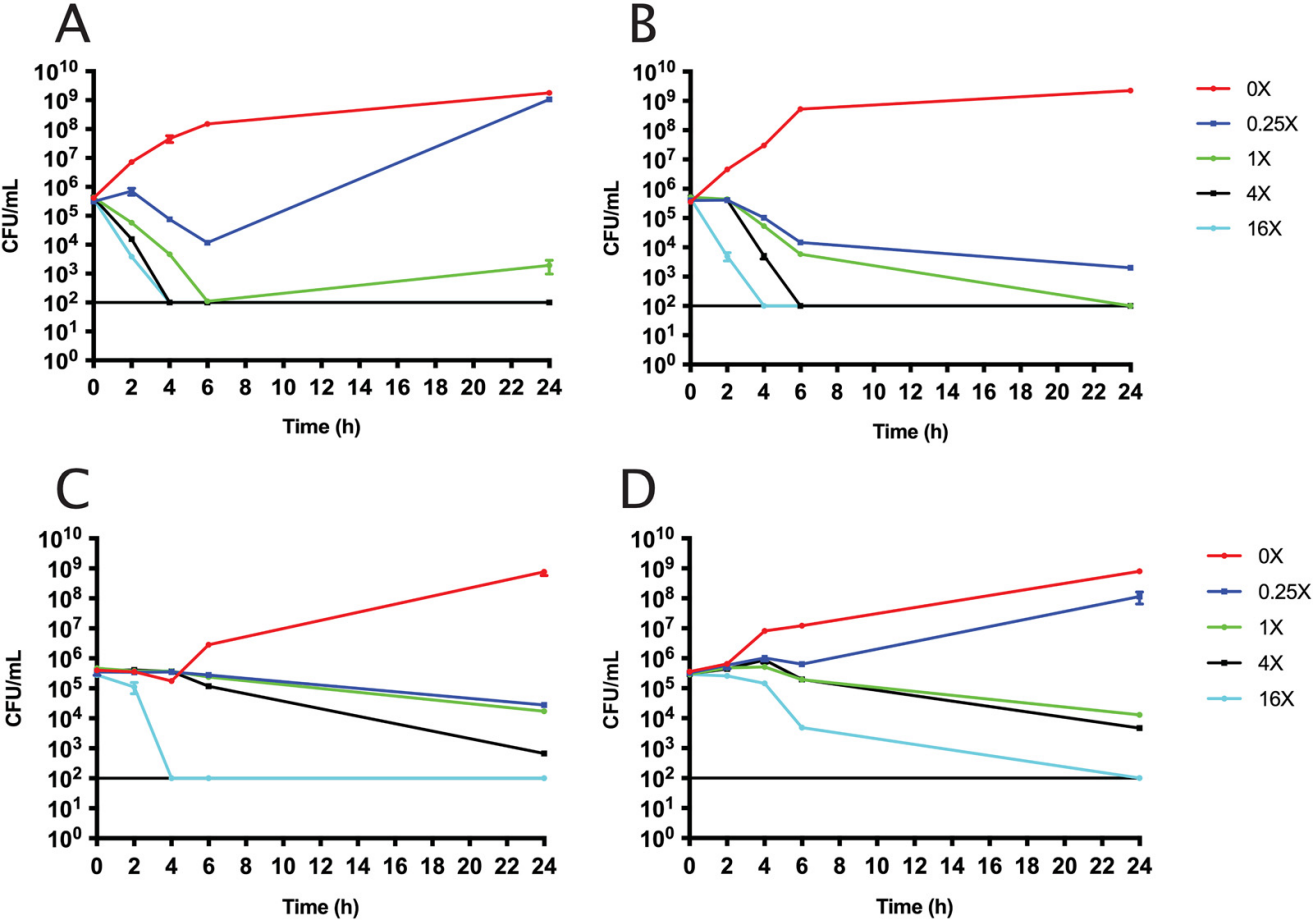
Time-kill curves for E. coli CDC-0001 cells treated with 0×, 0.25×, 1×, 4×, or 16× MIC of cefepime or cefepime-taniborbactam (*N* = 3 for all data points). Plotted are the CFU/ml values after hours of antibiotic treatment at 37°C with cefepime alone (1× MIC: 128 μg/ml) (A) or cefepime with 4 μg/ml taniborbactam (1× MIC: 4 μg/ml) (B). The same time-kill curves were generated after treatment at room temperature with cefepime alone (1× MIC: 128 μg/ml) (C) or cefepime with 4 μg/ml taniborbactam (1× MIC: 4 μg/ml) (D). The horizontal line on each graph indicates the lower limit of detection.

Time-kill assays were performed with E. coli CDC-0001 (Fig. 1) to examine whether the addition of taniborbactam affects the rate of killing by cefepime. The results of these assays showed a concentration-dependent killing for both treatments at 37°C (Fig. 1A and B) and at room temperature (Fig. 1C and D). Cell killing by cefepime occurred quickly at 37°C, with CFU falling below the limit of detection (10^2^ CFU/ml) after 4 h of treatment with 16× MIC of cefepime with or without 4 μg/ml taniborbactam. Given that all subsequent experiments were performed at room temperature, the following discussion highlights the differences seen between Fig. 1C and D. When comparing the rate of killing at 16× MIC by cefepime alone (2,048 μg/ml) versus cefepime plus taniborbactam (64 μg/ml cefepime plus 4 μg/ml taniborbactam), cefepime alone resulted in a greater extent of cell killing at 4 h. There are at least two related explanations for this observation. First, initial periplasmic cefepime concentrations may be higher in the cefepime alone condition simply due to the different dosing concentration (2,048 μg/ml versus 64 μg/ml). Second, initial periplasmic cefepime concentrations may be higher in the cefepime alone conditions due to parameters of kinetic turnover of cefepime by KPC-3. While the *K_m_* and *k*_cat_ of KPC-3 are not known, KPC-2 differs from KPC-3 by only one amino acid substitution (H272Y) and has a *K_m_* of >1,000 μM and a *k*_cat_ of >6 s^−1^ (14, 15). These values suggest that in the first 2 h, cefepime may be slowly sequestered by binding to KPC-3 without hydrolysis. If the initial cefepime concentration is higher, it is more likely that cefepime would saturate the binding sites of KPC-3 proteins and that excess cefepime would irreversibly inhibit both PBP3 and other PBP targets, which would induce rapid lysis.

### Bacterial morphology with antibiotic treatments.

**FIG 2:**
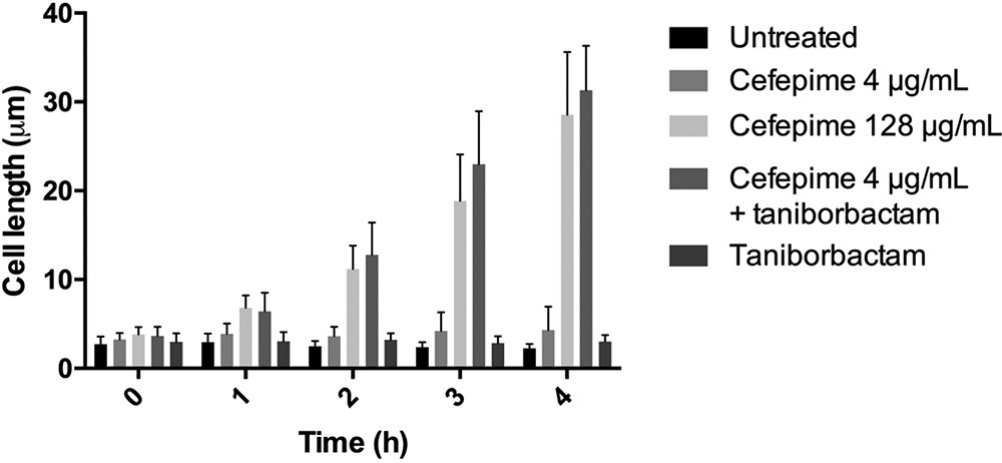
Average length of E. coli CDC-0001 cells untreated or treated with 4 μg/ml cefepime, 128 μg/ml cefepime, 4 μg/ml cefepime + 4 μg/ml taniborbactam, and 4 μg/ml taniborbactam over time. Error bars show the standard deviations.

PBP3 is integral to bacterial cell division, and, therefore, inhibition results in a septation defect with continuous elongation inducing cell filamentation (16–18). To monitor the effect of these PBP inhibitors on PBP3, we measured resulting changes in cell length by time-lapse microscopy. Because the trend of the time-kill curves at room temperature was similar to that at 37°C (Fig. 1), we observed growth and filamentation of single cells at room temperature. The average length of cells (30 ≤ *N* ≤ 100) was determined from images taken every hour for 4 h at 0× and 1× MIC for cefepime in the presence of 4 μg/ml taniborbactam (Fig. 2). To monitor the phenotype of E. coli CDC-0001 cells treated with cefepime alone, cells were imaged following treatment with 4 μg/ml and 128 μg/ml cefepime (concentrations equivalent to 1× MIC cefepime plus taniborbactam and 1× MIC of cefepime alone, respectively). After exposure to cefepime-taniborbactam (4 μg/ml each) and cefepime alone (128 μg/ml), cells exhibited significant elongation at 1 h posttreatment and by 4 h reached a maximum length of 32 ± 5 μm and 29 ± 7 μm, respectively. Cells treated with 4 μg/ml cefepime alone or 4 μg/ml taniborbactam alone did not elongate and reached maximum average lengths of 4.3 ± 2.6 μm and 3.7 ± 1.6 μm, respectively. Overall, these results provide visual confirmation that taniborbactam restores antibacterial activity of cefepime at 4 μg/ml against KPC-3-producing E. coli CDC-0001.

**FIG 3:**
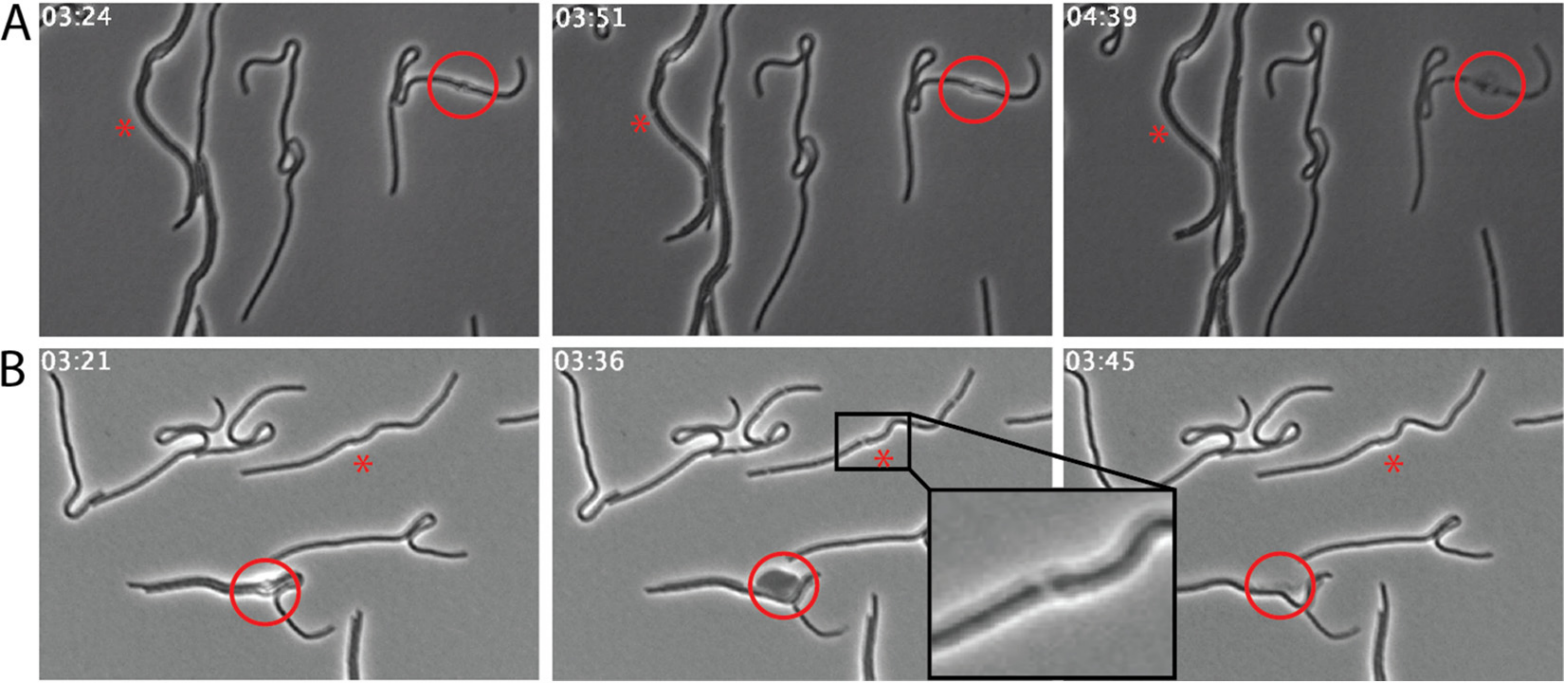
Individual images taken from time-lapse videos of E. coli CDC-0001 cells treated with 1× MIC (0.25 μg/ml) aztreonam (A) and 1× MIC cefepime + 4 μg/ml taniborbactam (B). Aztreonam-treated cells act as a control in this experiment, demonstrating typical morphological effects of a β-lactam on E. coli. For cells treated with cefepime + 4 μg/ml taniborbactam, cellular voids (marked with an asterisk [*]) appeared in 62% ± 9% of the population, and these voids appeared and disappeared within 12 ± 10 min (*N* = 15) (low magnification fields of view used for image analysis are shown in Movie S5 in the supplemental material). The red circles highlight a point of cell bulging followed by lysis at that same point. The time after treatment of cefepime is shown in the top-left corner of each image (h:min).

Time-lapse videos of individual cells treated with antibiotics were generated to evaluate morphological changes elicited from antibiotic exposure. E. coli CDC-0001 cells were treated with 1×, 2×, and 4× MIC of cefepime combined with 4 μg/ml taniborbactam (Movie S1). As controls, time-lapse microscopy experiments were also performed with cells treated with 4, 8, or 16 μg/ml cefepime alone or 4 μg/ml taniborbactam alone (Movies S2 and S3). Treatment of cells with 1×, 2×, and 4× MIC cefepime combined with 4 μg/ml taniborbactam resulted in elongated cells, subsequent cessation of growth, and bulge formation at the midpoint after 2 to 3 h of treatment (Fig. 3B). These cells exhibited midcell bulges leading to lysis from disruption of the cell envelope. Notably, treatment with 1× MIC cefepime combined with 4 μg/ml taniborbactam induced a phenotype exhibiting an apparent cellular void (Fig. 3B), which consistently appeared and disappeared within 12 ± 10 min. Comparable images of cells treated with aztreonam were obtained as a control (Fig. 3A). Aztreonam, a PBP3-specific β-lactam antibiotic, was used as a control in order to establish a baseline morphological effect of an antibiotic acting on PBP3. The morphological changes observed for both treatments are consistent with a disruption in cell division caused by inhibition of PBP3. For cells treated with cefepime-taniborbactam, voids occurred in 62% ± 9% of cells and could be the result of inner membrane pinching at initiated division sites, where the constrictive force is not enough to contract the cell wall and outer membrane layers. Similar cellular voids have been described in relation to a nonfunctional cell division protein variant, FtsK (19).

Cells treated with various concentrations of cefepime alone exhibited differing extents of elongation and cell morphologies. The treatment with 4 μg/ml cefepime (1/32× MIC) initially resulted in slightly elongated cells, which then returned to a regular pattern of cellular division (Movie S2). Cells treated with 8 μg/ml and 16 μg/ml (1/16× MIC and 1/8× MIC) cefepime elongated significantly without cell lysis or any cessation of growth (Movie S2). These treatments also resulted in the same apparent cellular voids as seen with the combined cefepime plus taniborbactam treatment (16 μg/ml, 4× MIC). As expected, an increasing propensity for elongation was observed at higher concentrations of cefepime. As bacteria produce a finite number of β-lactamases, their effect can be overwhelmed at elevated β-lactam concentrations. As anticipated from its lack of standalone antibacterial activity, taniborbactam-treated cells grew and divided normally even at 16 μg/ml (Movie S3).

**FIG 4:**
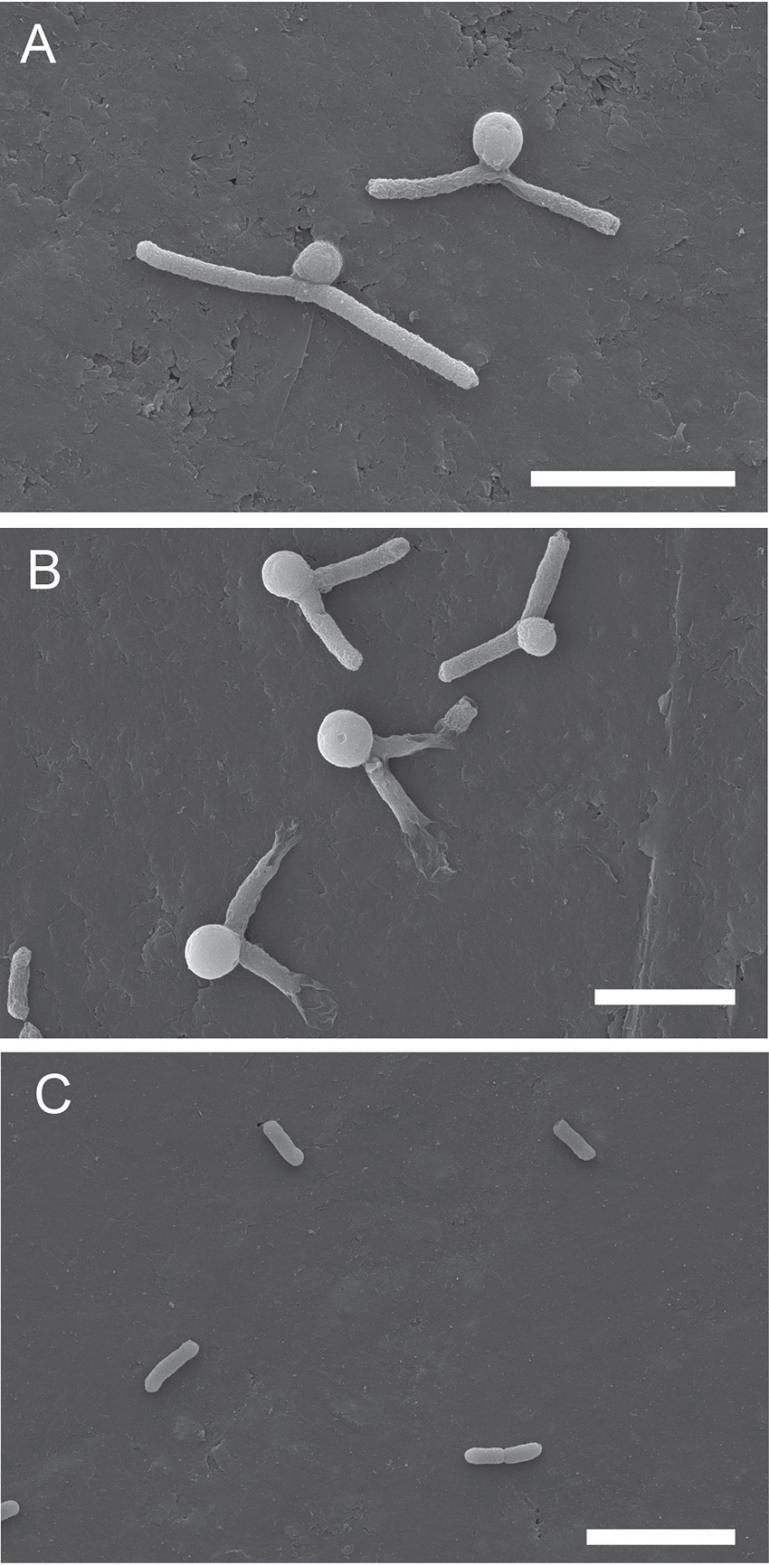
Scanning electron micrographs of CDC-0001 cells treated for 4 h with 16 μg/ml cefepime + 4 μg/ml taniborbactam (A) and with 128 μg/ml cefepime (B) compared to untreated cells (C). Cell elongation and membrane bulging were observed in cells treated with 16 μg/ml cefepime + 4 μg/ml taniborbactam and in cells treated with 128 μg/ml cefepime. Collapse at the poles of cells with large center cell bulges was observed in cells treated with 128 μg/ml cefepime. These collapsed poles were not observed consistently in any live microscopy sample and are likely due to dehydration during sample preparation, a common artifact in electron microscopy; scale bars = 5 μm.

Treatment with either 16 μg/ml cefepime combined with 4 μg/ml taniborbactam (4× MIC) or 128 μg/ml cefepime alone (1× MIC) commonly resulted in large midcell bulges, indicating a weakness in the cell wall at the initiated division site (Fig. 3). These same cells, when viewed in time-lapse images by phase microscopy, lysed within minutes after the midcell bulges formed (Fig. 3B). Scanning electron microscopy (SEM) analysis of these samples revealed that after 4 h of treatment, cells showed similar midcell bulging with 4× MIC cefepime combined with 4 μg/ml taniborbactam (Fig. 4A) and with 1× MIC cefepime alone (128 μg/ml) (Fig. 4B) compared to what was observed in untreated cells (Fig. 4C). Because cefepime has high affinity to PBP3 but also binds PBP2 and PBP1s (9), cefepime at high concentrations inhibits multiple PBPs, leading to cell lysis, while cefepime induces elongation by inhibiting PBP3 predominantly at low concentrations. This further supports initial findings from time-kill assays where cell death occurred more rapidly in the presence of cefepime at higher multiples of MIC, allowing it to inhibit multiple PBPs simultaneously.

### Taniborbactam elicits a lasting effect of cefepime.

Taniborbactam has been previously reported to inhibit KPC-2 β-lactamase with a half-life of 105 min (6). Here, experiments were designed to wash away excess taniborbactam after a pretreatment of the cells. This allowed us to specifically monitor the time course of morphological recovery to rod shape by release of taniborbactam from the KPC-3 active site and subsequent restoration of cefepime hydrolysis by KPC-3. We first treated E. coli CDC-0001 cells with 1× MIC cefepime (4 μg/ml) combined with 4 μg/ml taniborbactam for 4 h, washed the cells, and then captured time-lapse images of cells every 3 min for 12 h. Cells that were observed at time zero of imaging elongated further, and cellular division resumed after ∼3.5 h (Movie S4). Almost all first divisions (89%) were at the poles of the cells (Table 2), in good agreement with previous reports (20). Subsequent divisions occurred predominantly at the poles of cells (67%) but with an increased proportion of cells exhibiting midcell divisions. This return to midcell division is likely a result of shorter cell length and proper placement of the cell division machinery. A similar phenomenon was also observed for cells treated with 128 μg/ml cefepime (i.e., 1× MIC) alone (Table 2).

**TABLE 2:** Polar cell division during recovery following antibiotic treatment

Treatment	Frequency of polar first division (*N* = 3^*a*^)	Frequency of polar second division (*N* = 3^*a*^)
Cefepime (4 μg/ml) + taniborbactam (4 μg/ml)	89% ± 7%	67% ± 6%
Cefepime (128 μg/ml)	78% ± 4%	69% ± 8%

a Three biological replicates recorded for greater than 25 cells per replicate.

**FIG 5:**
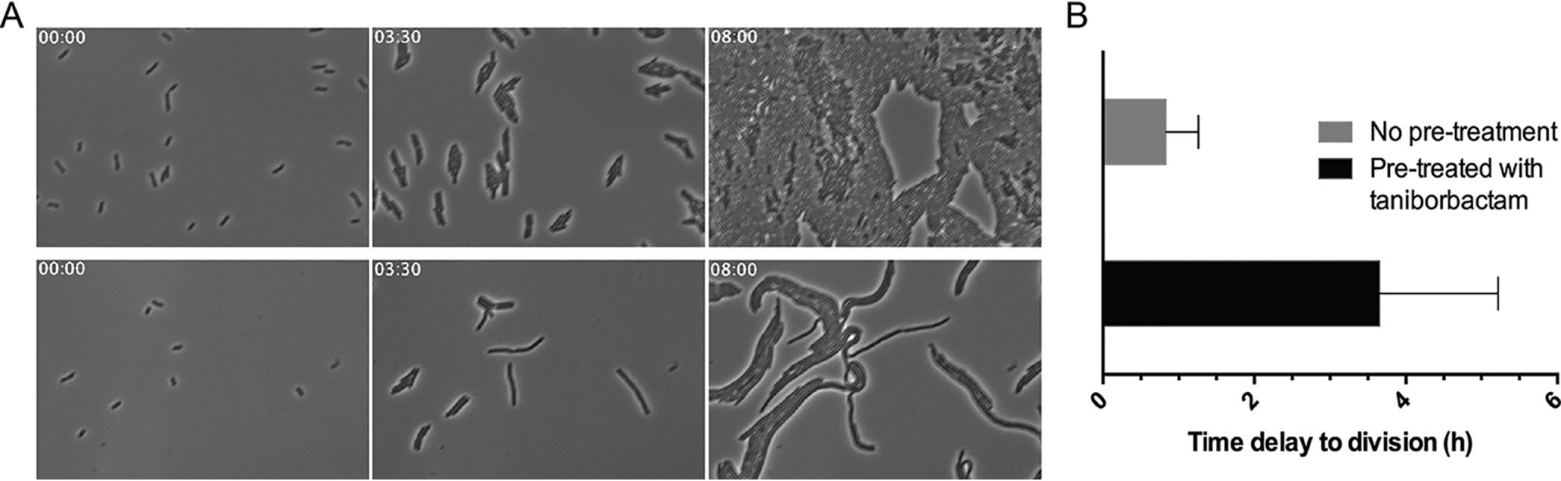
Post-β-lactamase inhibitor effect of taniborbactam. (A) Shown are individual images from time-lapse videos of KPC-3-producing E. coli CDC-0001 cells exposed to 4 μg/ml cefepime without (top) or with pretreatment with 4 μg/ml taniborbactam (bottom). (B) The average time periods taken for occurrence of the first cell division events (*N* = 42) in untreated or taniborbactam-treated cells grown with 4 μg/ml cefepime were measured by image analysis and plotted with standard deviations (error bars). The delay of the first cell division events by pretreatment with taniborbactam is significant (*P* < 0.0005). The time after treatment of cefepime is shown in the top-left corner of each image (h:min).

In the second recovery assay, cells were pretreated with 4 μg/ml taniborbactam for 4 h, after which the taniborbactam was washed away and cefepime was added at 4 μg/ml (Fig. 5A, bottom; Movie S4). As a control, cells were not pretreated with taniborbactam prior to treatment with 4 μg/ml cefepime (Fig. 5A, top). Thus, normal rod cells were observed at time zero of imaging in the experiments. While cells without pretreatment with taniborbactam began to divide after 21 min, cells pretreated with taniborbactam exhibited a significant (*P* < 0.0005) delay in division and began to divide after ∼4 h (Fig. 5B). This delayed return to normal cell division likely reflects a combination of new synthesis of KPC-3 and PBPs and slow release of taniborbactam from the KPC-3-taniborbactam complex. The results from both of these experiments are consistent with a long residency time of taniborbactam within the active site of KPC-3 (6) in E. coli cells and provides prolonged protection from KPC-3 for cefepime to exhibit its antibacterial activity.

## CONCLUSION

In this study, we investigated the morphological effects of cefepime-taniborbactam on KPC-3-producing E. coli CDC-0001 and the post-BLI effects on cefepime after pretreatment with taniborbactam. Taniborbactam was successful in inhibiting the KPC-3 β-lactamase and shifting the MIC of cefepime from 128 μg/ml alone to 4 μg/ml in the presence of taniborbactam. Treating E. coli CDC-0001 with the cefepime-taniborbactam combination resulted in cell death, morphological defects, and physical lysis, consistent with the controls of aztreonam and cefepime at a much higher concentration. When taniborbactam was used as a pretreatment, it exhibited a lasting protective effect, enabling the antibacterial activity of the cefepime in E. coli. This result confirms the long residency time of taniborbactam in KPC-3, consistent with the previously studied KPC-2 (6).

## MATERIALS AND METHODS

### Strains, compounds, and bacterial growth.

E. coli CDC-0001 (BioSample accession number SAMN04014842) was used in this study. Unless otherwise stated, bacterial cultures were grown at 37°C in cation-adjusted Mueller-Hinton broth (CAMHB) (Fisher Scientific) in the presence or absence (negative controls) of taniborbactam (Venatorx Pharmaceuticals, Inc.) or cefepime hydrochloride (USP).

### MIC assays.

MIC values (Fig. S1 in the supplemental material) were determined using the method described by Wiegand and colleagues and produced MIC results equivalent to the CLSI M07 edition 11 (2018) broth microdilution method (21, 22). The bacterial strain used also produced results consistent with the quality control ranges stated in the CLSI M100 edition 30 document (23). Briefly, the conversion factor to obtain a CFU/ml based on an optical density at 600 nm (OD_600_) was determined experimentally (an OD_600_ of 1 was equal to 7.2 × 10^8^ CFU/ml). An overnight culture was diluted to a final inoculum of 5 × 10^5^ CFU/ml in each test well along with the appropriate serially diluted antibiotic to be tested. Each sample was run in technical quadruplicate and biological triplicate, and MICs were determined using the mode of the concentration at which there was a 90% reduction in cell turbidity compared to the growth control, as determined using a spectrophotometer. These experiments were performed in flat-bottom polystyrene 96-well plates.

### Time-kill assays.

The procedure for determining the time it takes for an antibiotic to kill bacterial cells is described in detail in the CLSI-approved guideline document M26-A (22). Briefly, cells were inoculated in liquid medium to 5 × 10^5^ CFU/ml with various concentrations of antibiotic, based on previously determined MICs (0.25×, 1×, 4×, and 16× MIC) along with an untreated control (0× MIC). These were then incubated for set amounts of time (0, 2, 4, 6, and 24 h) and assessed for viability by diluting serially 10-fold and spot plating each dilution of the cultures on antibiotic-free agar plates. These experiments were conducted in technical and biological triplicates.

### Time-lapse microscopy.

Time-lapse images were taken by using NIS-Elements and an Eclipse Ti2 microscope (Nikon) equipped with a ×40 long working distance objective and an Orca Flash 4.0 LT camera (Hamamatsu). An overnight culture of E. coli CDC-0001 was diluted 100-fold in fresh CAMHB and grown at 30^°^C to an OD_600_ of 0.4. Cells were then diluted 2-fold in medium containing antibiotics. These treated cells were then placed in an 8-chamber slide (Thermo Scientific Nunc Lab-Tek II chambered coverglass) and covered with a nutrient agarose pad (1%) containing the same desired concentration of antibiotic (0×, 1×, 2×, or 4× MIC). The nutrient agarose pad was topped with a small piece of coverglass and sealed at the top with Breathe-Easy polyurethane moisture barrier (Diversified Biotech). The 8-chamber slides were then imaged every 3 min for each time-lapse experiment. Images were then compiled into videos at 7 frames per second in ImageJ (24).

### Cell length measurements.

Snapshots taken from time-lapse experiments were analyzed to track cell lengths under different treatment conditions over time. For the time-lapse experiments, cells were monitored at room temperature during treatment. All cells in a field of view were measured using ImageJ software to a maximum *N* of 100 every hour for 4 h. Data were compiled in Prism, and two-way analysis of variance (ANOVA) was performed comparing each test data point to the control data point at the corresponding time point.

### Scanning electron microscopy.

Samples of cells for scanning electron microscopy (SEM) were grown up to an OD_600_ of 0.4 from the diluted overnight culture. Cells were then diluted 2-fold into medium containing antibiotics and incubated for 4 h. Samples were then adhered to carbon planchets and fixed with glutaraldehyde (2%) and osmium tetroxide (1%) followed by an ethanol dehydration series. Samples were then fully dried using a Denton DCP-1 critical point dryer and were sputter-coated in gold-palladium using a Denton Desk V TSC. Samples were then imaged using a Quanta FEG 250 SEM operated at 10 kV.

### Recovery assays.

Recovery assays were set up similar to the time-lapse experiments with a few modifications. After dilution and the addition of antibiotics, cells at log phase were incubated in a 1.5-ml microcentrifuge tube for 4 h in the absence or presence of antibiotic. After this treatment, cells were pelleted and resuspended in fresh CAMHB and imaged in the same 8-chamber slide setup as for the time-lapse experiments. Samples were imaged every 3 min for 12 h.

## References

[1] CDC. 2019. Antibiotic resistance threats in the United States. U.S. Department of Health and Human Services, CDC, Atlanta, GA.

[2] Jean SS, Gould IM, Lee WS, Hsueh PR, International Society of Antimicrobial Chemotherapy (ISAC). 2019. New drugs for multidrug-resistant Gram-negative organisms: time for stewardship. Drugs 79:705–714. doi:10.1007/s40265-019-01112-1. 30972660

[3] Drawz SM, Bonomo RA. 2010. Three decades of beta-lactamase inhibitors. Clin Microbiol Rev 23:160–201. doi:10.1128/CMR.00037-09. 20065329 PMC2806661

[4] Martínez-Martínez L, González-López JJ. 2014. Carbapenemases in *Enterobacteriaceae*: types and molecular epidemiology. Enferm Infecc Microbiol Clin 32:4–9. doi:10.1016/S0213-005X(14)70168-5. 25542046

[5] Lomovskaya O, Sun D, Rubio-Aparicio D, Nelson K, Tsivkovski R, Griffith DC, Dudley MN. 2017. Vaborbactam: spectrum of β-lactamase inhibition and impact of resistance mechanisms on activity in *Enterobacteriaceae*. Antimicrob Agents Chemother 61:e01443-17. doi:10.1128/AAC.01443-17. 28848018 PMC5655098

[6] Hamrick JC, Docquier J-D, Uehara T, Myers CL, Six DA, Chatwin CL, John KJ, Vernacchio SF, Cusick SM, Trout REL, Pozzi C, De Luca F, Benvenuti M, Mangani S, Liu B, Jackson RW, Moeck G, Xerri L, Burns CJ, Pevear DC, Daigle DM. 2020. VNRX-5133 (taniborbactam), a broad-spectrum inhibitor of serine- and metallo-β-lactamases, restores activity of cefepime in *Enterobacterales* and *Pseudomonas aeruginosa*. Antimicrob Agents Chemother 64:e01963-19. doi:10.1128/AAC.01963-19. 31871094 PMC7038240

[7] Liu B, Trout REL, Chu G-H, McGarry D, Jackson RW, Hamrick JC, Daigle DM, Cusick SM, Pozzi C, De Luca F, Benvenuti M, Mangani S, Docquier J-D, Weiss WJ, Pevear DC, Xerri L, Burns CJ. 2020. Discovery of taniborbactam (VNRX-5133): a broad-spectrum serine- and metallo-β-lactamase inhibitor for carbapenem-resistant bacterial infections. J Med Chem 63:2789–2801. doi:10.1021/acs.jmedchem.9b01518. 31765155 PMC7104248

[8] Wang X, Zhao C, Wang Q, Wang Z, Liang X, Zhang F, Zhang Y, Meng H, Chen H, Li S, Zhou C, Li H, Wang H. 2020. *In vitro* activity of the novel β-lactamase inhibitor taniborbactam (VNRX-5133), in combination with cefepime or meropenem, against MDR Gram-negative bacterial isolates from China. J Antimicrob Chemother 75:1850–1858. doi:10.1093/jac/dkaa053. 32154866

[9] Pucci MJ, Boice-Sowek J, Kessler RE, Dougherty TJ. 1991. Comparison of cefepime, cefpirome, and cefaclidine binding affinities for penicillin-binding proteins in *Escherichia coli* K-12 and *Pseudomonas aeruginosa* SC8329. Antimicrob Agents Chemother 35:2312–2317. doi:10.1128/AAC.35.11.2312. 1804003 PMC245377

[10] Uehara T, Bernhardt TG. 2011. More than just lysins: peptidoglycan hydrolases tailor the cell wall. Curr Opin Microbiol 14:698–703. doi:10.1016/j.mib.2011.10.003. 22055466 PMC3347972

[11] Marshall WF, Blair JE. 1999. The cephalosporins. Mayo Clin Proc 74:187–195. doi:10.4065/74.2.187. 10069359

[12] Yao Z, Kahne D, Kishony R. 2012. Distinct single-cell morphological dynamics under β-lactam antibiotics. Mol Cell 48:705–712. doi:10.1016/j.molcel.2012.09.016. 23103254 PMC3525771

[13] Hammoudi Halat D, Ayoub Moubareck C. 2020. The current burden of carbapenemases: review of significant properties and dissemination among Gram-negative bacteria. Antibiotics (Basel) 9:186. doi:10.3390/antibiotics9040186. 32316342 PMC7235769

[14] Bush K, Bradford PA. 2020. Epidemiology of β-lactamase-producing pathogens. Clin Microbiol Rev 33:e00047-19. doi:10.1128/CMR.00047-19. 32102899 PMC7048014

[15] Levitt PS, Papp-Wallace KM, Taracila MA, Hujer AM, Winkler ML, Smith KM, Xu Y, Harris ME, Bonomo RA. 2012. Exploring the role of a conserved class A residue in the Ω-loop of KPC-2 β-lactamase: a mechanism for ceftazidime hydrolysis. J Biol Chem 287:31783–31793. doi:10.1074/jbc.M112.348540. 22843686 PMC3442512

[16] Nguyen-Distèche M, Fraipont C, Buddelmeijer N, Nanninga N. 1998. The structure and function of *Escherichia coli* penicillin-binding protein 3. Cell Mol Life Sci 54:309–316. doi:10.1007/s000180050157. 9614966 PMC11147360

[17] Marmont LS, Bernhardt TG. 2020. A conserved subcomplex within the bacterial cytokinetic ring activates cell wall synthesis by the FtsW-FtsI synthase. Proc Natl Acad Sci USA 117:23879–23885. doi:10.1073/pnas.2004598117. 32907942 PMC7519343

[18] Typas A, Banzhaf M, Gross CA, Vollmer W. 2011. From the regulation of peptidoglycan synthesis to bacterial growth and morphology. Nat Rev Microbiol 10:123–136. doi:10.1038/nrmicro2677. 22203377 PMC5433867

[19] Berezuk AM, Goodyear M, Khursigara CM. 2014. Site-directed fluorescence labeling reveals a revised N-terminal membrane topology and functional periplasmic residues in the *Escherichia coli* cell division protein FtsK. J Biol Chem 289:23287–23301. doi:10.1074/jbc.M114.569624. 25002583 PMC4156081

[20] Wehrens M, Ershov D, Rozendaal R, Walker N, Schultz D, Kishony R, Levin PA, Tans SJ. 2018. Size laws and division ring dynamics in filamentous *Escherichia coli* cells. Curr Biol 28:972–979. doi:10.1016/j.cub.2018.02.006. 29502951

[21] Wiegand I, Hilpert K, Hancock RE. 2008. Agar and broth dilution methods to determine the minimal inhibitory concentration (MIC) of antimicrobial substances. Nat Protoc 3:163–175. doi:10.1038/nprot.2007.521. 18274517

[22] CLSI. 2018. Methods for dilution antimicrobial susceptibility tests for bacteria that grow aerobically, 11th ed. Approved Guideline, CLSI document M07. Clinical and Laboratory Standards Institute, Wayne, PA.

[23] CLSI. 2020. Performance standards for antimicrobial susceptibility testing, 30th ed. Approved Guideline, CLSI document M100. Clinical and Laboratory Standards Institute, Wayne, PA.

[24] Schneider CA, Rasband WS, Eliceiri KW. 2012. NIH Image to ImageJ: 25 years of image analysis. Nat Methods 9:671–675. doi:10.1038/nmeth.2089. 22930834 PMC5554542

