# Supplementary material for: The Next-Generation β-Lactamase Inhibitor Taniborbactam Restores the Morphological Effects of Cefepime in KPC-Producing Escherichia coli
Source: Microbiol Spectr. 2021 Sep 8;9(2):e00918-21. doi: 10.1128/Spectrum.00918-21 (PMC8557880; doi:10.1128/Spectrum.00918-21)
Supplement: SUPPLEMENTAL FILE 6 — Supplemental material. Download SPECTRUM00918-21_Supp_1_seq2.docx, DOCX file, 0.1 MB [file spectrum00918-21_supp_1_seq2.docx]

**SUPPLEMENTAL DATA**

The next-generation β-lactamase inhibitor taniborbactam restores the morphological effects of cefepime in KPC-producing *Escherichia coli*

Elyse J. Roach,^a^ Tsuyoshi Uehara,^b^ Denis M. Daigle,^b^ David A. Six,^b^ Cezar M. Khursigara^a^

^a^Department of Molecular and Cellular Biology, University of Guelph, Guelph, Ontario, Canada

^b^Venatorx Pharmaceuticals, Inc., Malvern, Pennsylvania, USA

Address correspondence to Cezar Khursigara, [ckhursig@uoguelph.ca](mailto:ckhursig@uoguelph.ca)

**
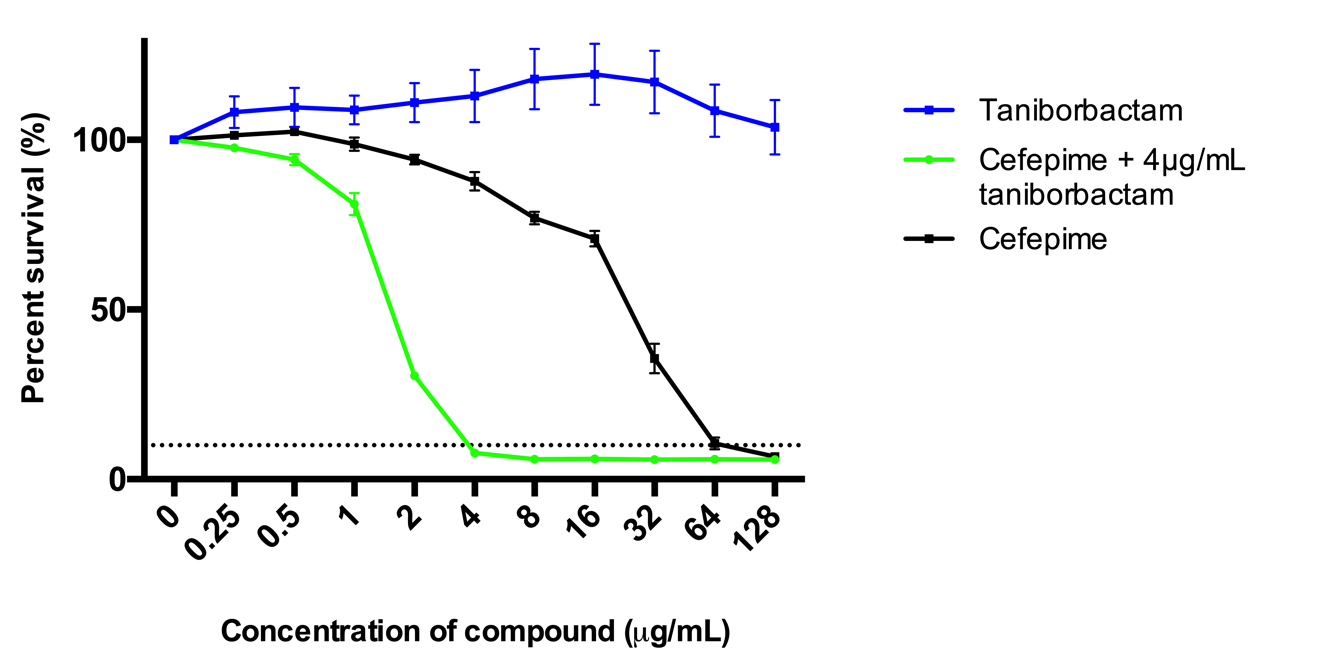
**

**Figure S1.** MIC data from *E. coli* CDC-0001 testing the susceptibility to taniborbactam alone, cefepime alone and cefepime potentiated with 4 µg/mL taniborbactam, compared to untreated control samples. The dotted line indicates 10% survival.

**Movie S1**. Time-lapse videos of *E. coli* CDC-0001 treated with cefepime + 4 µg/mL taniborbactam at 1× MIC (left panel), 2× MIC (middle panel) and 4× MIC (right panel).

**Movie S2.** Time-lapse videos of *E. coli* CDC-0001 treated with cefepime at 4 µg/mL (left panel), 8 µg/mL (middle panel) and 16 µg/mL (right panel).

**Movie S3**. Time-lapse videos of *E. coli* CDC-0001 treated with taniborbactam at 4 µg/mL (left panel), 8 µg/mL (middle panel) and 16 µg/mL (right panel).

**Movie S4**. Time-lapse videos of *E. coli* CDC-0001 grown with 4 µg/mL cefepime without or with pre-treatment with taniborbactam or cefepime plus taniborbactam. The left panel shows a sample which had no pre-treatment prior to incubation with 4 µg/mL cefepime, and the middle panel shows cells pre-treated with 4 µg/mL taniborbactam which was washed away prior to incubation with 4 µg/mL cefepime. The right panel shows a sample pre-treated with 4 µg/mL cefepime and 4 µg/mL taniborbactam which were washed away prior to treatment with 4 µg/mL cefepime. Cells were imaged upon treatment with cefepime.

**Movie S5**. Time-lapse videos of *E. coli* CDC-001 treated with 1× MIC cefepime and 4 µg/mL taniborbactam at low magnification, used for statistical analysis as described in Figure 3.
